# Overexpression of eukaryotic initiation factor 5A (eIF5A) affects susceptibility to benznidazole in *Trypanosoma cruzi* populations

**DOI:** 10.1590/0074-02760180162

**Published:** 2018-07-26

**Authors:** Douglas de Souza Moreira, Ana Paula Duarte, Fabiano Sviatopolk Mirsky Pais, Rosiane Aparecida da Silva-Pereira, Alvaro José Romanha, Sergio Schenkman, Silvane Maria Fonseca Murta

**Affiliations:** 1 Fundação Oswaldo Cruz-Fiocruz, Instituto René Rachou, Belo Horizonte, MG, Brasil; 2 Universidade Federal de São Paulo, Departamento de Microbiologia, Imunologia e Parasitologia, São Paulo, SP, Brasil

**Keywords:** Trypanosoma cruzi, drug resistance, benznidazole, eukaryotic initiation factor 5A (eIF5A)

## Abstract

Eukaryotic initiation factor 5A (eIF5A) is a conserved protein with an essential role in translation elongation. Using one and two-dimensional western blotting, we showed that the eIF5A protein level was 2-fold lower in benznidazole (BZ)-resistant (BZR and 17LER) *Trypanosoma cruzi* populations than in their respective susceptible counterparts (BZS and 17WTS). To confirm the role of eIF5A in BZ resistance, we transfected BZS and 17WTS with the wild-type eIF5A or mutant eIF5A-S2A (in which serine 2 was replaced by alanine). Upon overexpressing eIF5A, both susceptible lines became approximately 3- and 5-fold more sensitive to BZ. In contrast, the eIF5A-S2A mutant did not alter its susceptibility to BZ. These data suggest that BZ resistance might arise from either decreasing the translation of proteins that require eIF5A, or as a consequence of differential levels of precursors for the hypusination reactions (e.g., spermidine and trypanothione), both of which alter BZ’s effects in the parasite.

The protozoan parasite *Trypanosoma cruzi* is the etiological agent of Chagas disease (American trypanosomiasis), which affects 6 to 7 million people worldwide, mainly in Latin America.^1^ Treatment consists of the administration of the drugs nifurtimox (5-nitrofuran; NFX) or benznidazole (2-nitroimidazole; BZ). Both compounds cause undesirable side effects and present low cure rates, mainly in the chronic phase of disease.^2^ NFX and BZ are prodrugs that need to be activated by nitroreductases to exert their trypanocidal effect. After reduction of NFX, nitro anion radicals react with oxygen, generating metabolites that are toxic to *T. cruzi*. BZ exerts its action via reductive stress, which causes deleterious effects on the DNA, proteins, and lipids of the parasite.^2^ Analysis of the genomes of three BZ-resistant *T. cruzi* clones showed that BZ metabolites have high mutagenic activity.^3^

Drug resistance is a serious public health problem in many countries. NFX and BZ resistance mechanisms in *T. cruzi* are still poorly understood, demonstrating the need of selecting new targets for Chagas disease chemotherapy. Several studies have identified different proteins, such as old yellow enzyme (NADPH dehydrogenase), iron-superoxide dismutase, tryparedoxin peroxidase, ascorbate peroxidase, hexose transporter, cyclophilin, lipoamide dehydrogenase, and aldo-keto reductase, which may contribute to BZ-resistance mechanisms in *T. cruzi*.^4^ ^-^ ^8^ Additionally, proteomic analyses have been used to better understand the mechanisms of drug resistance in various protozoan parasites. Interestingly, using two-dimensional electrophoresis combined with mass spectrometry, our group demonstrated that eukaryotic initiation factor 5A (eIF5A) was differentially expressed in trypomastigote forms from a BZ-resistant population (BZR) compared with that in its respective susceptible counterpart (BZS) (unpublished observations).

eIF5A is a highly conserved protein in eukaryotic organisms from archaebacteria to mammals. This factor participates in apoptosis, cell cycle control, and RNA decay. It was initially identified as a translation initiation factor; however, more recent studies revealed its role in translation elongation and termination.^9^ It acts by facilitating the elongation of polyproline, glycine, and charged amino acid-containing sequences.^10^ eIF5A is subjected to a posttranslational modification called hypusination, in which a hypusine residue, originating from spermidine, is added to a specific lysine residue. Thus, it is the only protein known to contain the uncommon amino acid residue hypusine, which is essential for the protein’s function. eIF5A and deoxyhypusine/hypusine modifications are essential for the growth of eukaryotic cells.^11^ eIF5A hypusination is believed to help peptide bond formation with structural constraints. Meanwhile non-modified eIF5A also appears to be required for the formation of other peptide bonds; in this case, in the presence of polyamines such as spermidine, which is the precursor of hypusine.^12^

In trypanosomes, eIF5A is essential for *Trypanosoma brucei* growth, possibly by preventing the synthesis of flagellar proteins, which are rich in polyproline residues.^13^ Furthermore, eIF5A phosphorylation and dephosphorylation at serine 2 seems to regulate growth, representing a crucial event for cell survival during stationary growth conditions.^14^ In contrast to most eukaryotes, trypanosomes have a different key molecule involved in redox metabolism called trypanothione, which comprises two glutathiones linked by a spermidine molecule.^15^ Polyamines are not synthesized by *T. cruzi* and are therefore rate limiting for parasite growth and survival, including their incorporation in trypanothione for redox metabolism and for the hypusination of eIF5A for translation activity.^13^

Therefore, in the present study, we determined whether the expression levels of eIF5A are related to the susceptibility to BZ of *T. cruzi* populations selected by gradual exposure to BZ.^16^ ^,^ ^17^ We also overexpressed the wild-type eIF5A or eIF5A-S2A mutant in BZ-susceptible *T. cruzi* populations.

eIF5A amino acid sequences are relatively conserved among trypanosomatids, with few modifications. An amino acid sequence alignment performed using an online version of MAFFT,^18^ and including the *Homo sapiens* eIF5A sequence, showed a high degree of conservation [Supplementary data, Figure (A)]. The evolutionary relationships among eIF5A sequences were inferred using the maximum likelihood method in PhyML^19^ (JTT substitution model; 100 bootstrapped data set) [Supplementary data, Figure (B)]. The phylogenetic analysis revealed a clear dichotomy between eIF5A sequences from trypanosomatids and that from *H. sapiens*. Despite the high similarity among all eIF5A trypanosomatids sequences, the tree topology clearly separated *Leishmania* from *Trypanosoma* species.

In the present study, we used a *T. cruzi* population selected *in vivo* for BZ resistance (BZR), which was derived from the Y strain, and its susceptible counterpart (BZS).^17^ These parasites were maintained in mice untreated (BZS) or treated (BZR) with a single high dose of BZ (500 mg/kg of body weight) at the peak of parasitaemia. The mice were bled after drug administration and the blood was seeded in liver infusion tryptose (LIT) medium to obtain *T. cruzi* epimastigote forms. A population with *in vitro*-induced BZ resistance (17LER) derived from the Tehuantepec cl2 strain and its susceptible counterpart (17WTS)^16^ were also included in this study. Epimastigote forms from these parasites were maintained in LIT medium at 28ºC.

Epimastigotes in the exponential growth phase (10^9^ parasites) were lysed in buffer containing 8 M urea, 2 M thiourea, 4% CHAPS (3-[(3-cholamidopropyl)dimethylammonio]-1-propanesulfonate), 50 mM dithiothreitol, 20 mM Tris-HCl, pH 7.4, and Complete Mini Protease Inhibitor Cocktail (Roche, Mannheim, Germany). After centrifugation at 20000 × *g* for 1 h, the supernatants (protein extracts) were quantified using the Bradford method and subjected to western blotting analysis using a specific antibody against *T. cruzi* eIF5A (anti-TceIF5A).^14^ The results showed that this antibody recognised an 18-kDa polypeptide, which is the expected size of eIF5A, in all *T. cruzi* samples analysed (Fig. 1A). The western blotting membranes were also incubated with anti-TcHSP70 (an antibody recognising *T. cruzi* heat shock protein 70), used as a normalisation antibody for the western blotting. Densitometry analysis showed that the eIF5A protein level was 2-fold lower in BZR and 17LER compared with that in their respective susceptible counterparts, BZS and 17WTS.

**Fig. 1:**
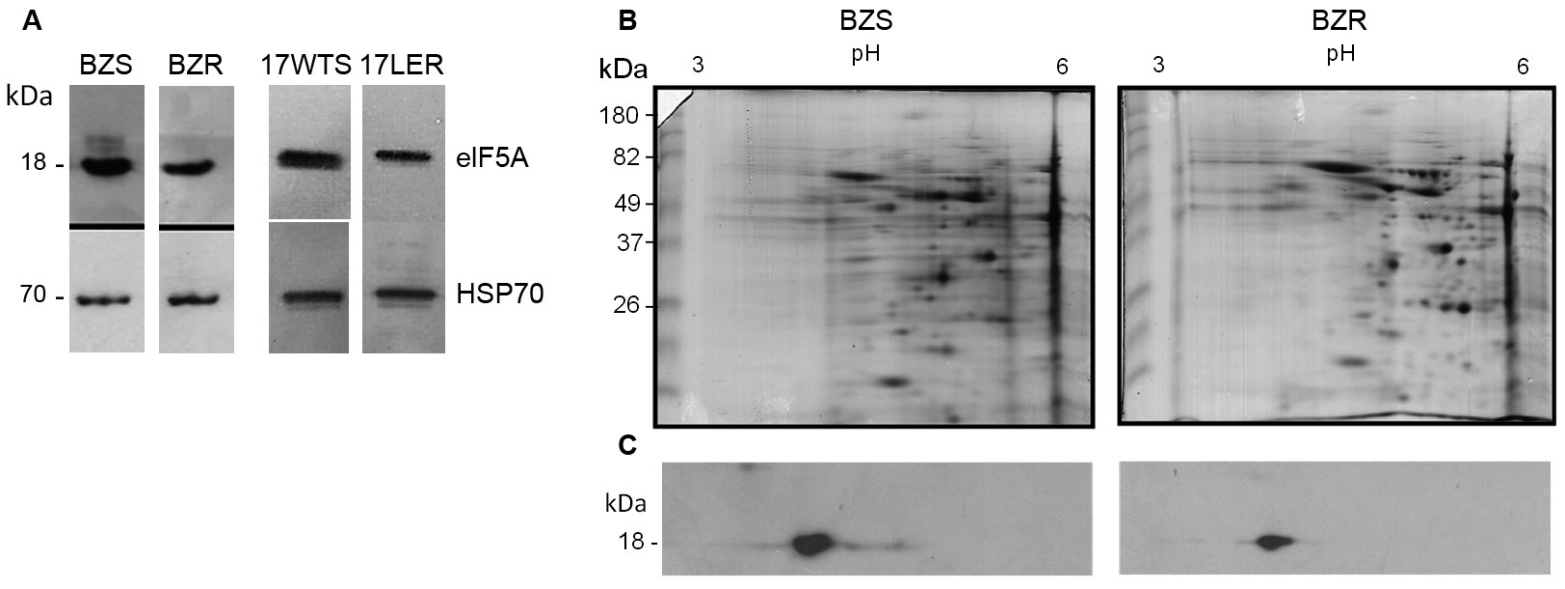
TceIF5A protein levels in benznidazole (BZ)-resistant *Trypanosoma cruzi* populations. (A) Western blotting of eIF5A. Extracts (2 µg) of epimastigotes from the samples BZS, BZR, 17WTS, and 17LER were subjected to electrophoresis on 12% SDS-polyacrylamide gels and blotted onto nitrocellulose membrane. The membranes were blocked, washed, and incubated for 1 h at 25ºC with the specific antibody recognising eIF5A from *T. cruzi* (1:500). The blots were washed and incubated with anti-mouse IgG conjugate labelled with horseradish peroxidase (HRP) (1:2000; Promega) for 30 min. After washing, the immunoreactive protein bands were revealed using chemiluminescence with an ECL Plus Kit (Amersham) according to the manufacturer’s protocol. To normalise the results, the membranes were incubated with polyclonal *T. cruzi* anti-TcHSP70 antibody. (B) Two-dimensional SDS-PAGE gels of proteins from BZS and BZR *T. cruzi* populations. Isoelectric focussing (IEF) was performed with 100 μg of protein using 7 cm, pH 3-6, immobilised pH gradient (IPG) strips. SDS-PAGE was performed on 12% polyacrylamide gels, which were stained with Coomassie blue G250. (C) Two-dimensional western blotting of BZS and BZR *T. cruzi* populations probed with anti-TceIF5A antibody according to the protocol described above.

We also performed two-dimensional electrophoresis (2DE) using the protocol described by Rêgo et al.^6^ The sodium dodecylsulphate polyacrylamide gel electrophoresis (SDS-PAGE) gels were stained with Colloidal Coomassie Blue G-250. Image analyses of the replicates obtained using a GS-800 scanner were performed to compare the BZ-susceptible and resistant samples (in triplicate). PDQuest 7.3. software (Bio-Rad) was used for the quantitative analysis of protein spot intensity and matching between the two-dimensional gels. Comparative analysis between Coomassie Blue-stained protein profiles of the BZS and BZR populations showed that loading of proteins was similar between these samples (Fig. 1B). 2DE western blotting results demonstrated that the anti-TceIF5A antibody recognised one spot with the expected molecular mass of 18 kDa and an isoelectric point (pI) around 4.5 in the both BZS and BZR (Fig. 1C). The intensity of the protein spot recognised by anti-TceIF5A was compared between BZS and BZR, and normalised using the same region of both Coomassie Blue-stained gels of these samples. This densitometric analysis showed that the eIF5A expression level was 2-fold lower in the BZR population compared with that in its susceptible counterpart BZS (Fig. 1C), corroborating the one-dimension western blotting result.

To investigate the role of *T. cruzi* eIF5A (TceIF5A) in resistance to BZ, 1 x 10^8^ parasites from the BZ-susceptible populations (BZS and 17WTS) were transfected with 100 μg of the plasmids p33-TceIF5A (expressing the wild-type TceIF5A) or p33-TceIF5A-S2A (expressing TceIF5A in which serine 2 was replaced by alanine).^14^ The transfection procedure followed the protocol described by da Rocha et al.^20^ Twenty-four hours post-transfection, parasites were placed under 250 μg/mL Geneticin G418 (Invitrogen) selection in LIT medium supplemented with 10% foetal bovine serum. This concentration of geneticin was maintained for three to four weeks and then increased to 500 μg/mL. Clonal lines resistant to this antibiotic were selected and analysed using PCR (with the primers forward 5′-CGTTGGCTACCCGTGATATT-3′ and reverse 5′-GCCCAGTCATAGCCGAATAG-3′) and western blotting to confirm the presence of the gene that confers resistance to geneticin and overexpression of eIF5A. Polymerase chain reaction (PCR) assays showed that all the geneticin-resistant clones presented a fragment of approximately 630 bp, which corresponded to a region of the neomycin phosphotransferase marker (data not shown). Western blotting showed that the anti-TceIF5A antibody recognised an 18-kDa protein in all samples analysed (Fig. 2A). Additionally, other polypeptide of approximately 25 kDa was also detected only in the BZS and 17WTS populations of *T. cruzi* transfected with plasmids p33-TceIF5A or p33-TceIF5A-S2A mutant. This could be a consequence of the overexpression of eIF5A protein in these samples or related to posttranslational modifications of eIF5A (Fig. 2A). The anti-TcHSP70 antibody showed that in all cases, the loading of proteins was similar among the *T. cruzi* populations evaluated (Fig. 2A).

Epimastigotes of BZS and 17WTS *T. cruzi* populations not transfected (BZS/17WTS Mock) or transfected with the plasmids p33-TceIF5A (BZS/17WTS eIF5A) or p33-TceIF5A-S2A (BZS/17WTS eIF5A-S2A mutant) were subjected to BZ susceptibility assays. Parasites were incubated in LIT medium at 2 × 10^6^ cells/mL in 24-well plates in the absence or presence of increasing BZ concentrations (2.5 to 20 μM) for 96 h. The effective concentration necessary to decrease growth by 50% (EC_50_) was determined using a model Z1 Coulter Counter (Beckman Coulter, Fullerton, CA, USA). EC_50_ values were obtained from three independent measurements in triplicate, using the linear interpolation method. The results showed that the BZ EC_50_ of untransfected BZS and 17WTS *T. cruzi* populations was 25 and 20 μM, respectively (Fig. 2B-C). By contrast, the BZS and 17WTS parasites transfected with eIF5A presented EC_50_ values of 8 μM and 4 μM, indicating that they became approximately 3- and 5-fold more susceptible to BZ than the wild-type BZS and 17WTS populations, respectively (Fig. 2B-C). Regarding transfection with eIF5A-S2A mutant protein, we observed that the susceptibility to BZ was not significantly altered in the transfected parasites compared with that of the non-transfected BZS and 17WTS populations, with BZ EC_50_ values for the mutant-transfected parasites of 20 μM and 19 μM, respectively (Fig. 2B-C).

eIF5A is a ubiquitous protein and is essential in all eukaryotic organisms. Some studies revealed that the expression of this enzyme correlated with different pathways in human diseases. The eIF5A expression level in human peripheral blood mononuclear cells increased in HIV-1 infected patients in comparison with uninfected people.^21^ Chen and Chen^22^ demonstrated that the hypusine formation activity increased in mouse cells transfected with a vector expressing the Ras oncogene. Overexpression of eIF5A2, one of the two isoforms in the eIF5A family, promotes tumour metastasis in hepatocellular carcinoma.^23^ Upregulated expression of eIF5A2 was also associated with poor survival of patients with gastric cancer.^24^ These results indicated that the levels of eIF5A lead to the different phenotypes observed.

**Fig. 2:**
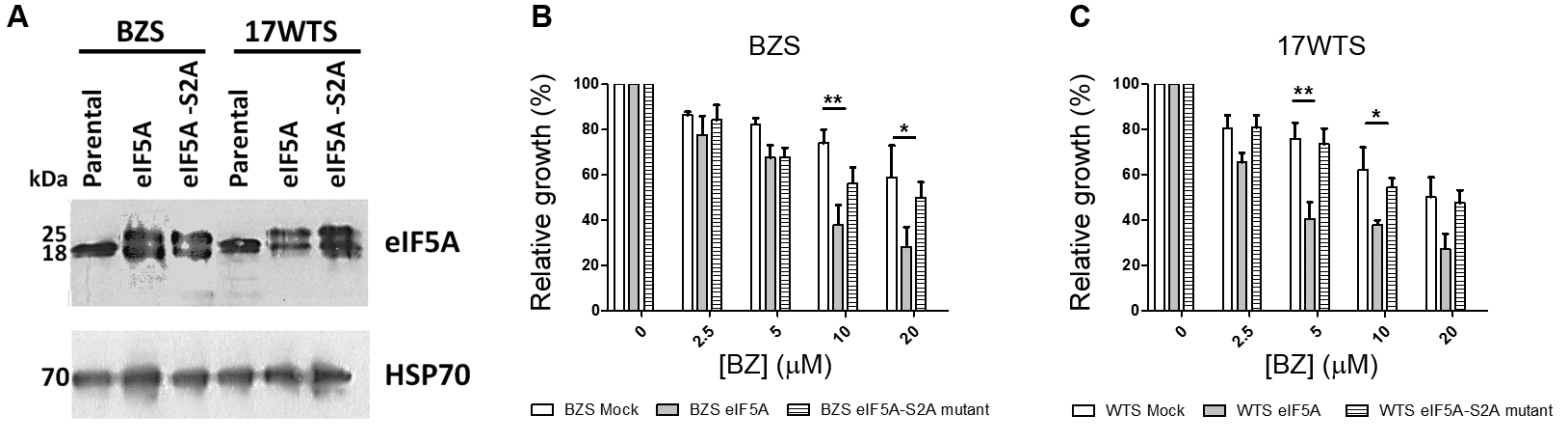
TceIF5A protein levels and susceptibility to benznidazole in BZS and 17WTS parasites overexpressing eIF5A or the eIF5A-S2A mutant protein. (A) Protein extracts of epimastigotes from BZS and 17WTS populations (60 µg) were electrophoresed using 12% SDS-PAGE and blotted onto nitrocellulose membranes. The blots were incubated with anti-TceIF5A antibody and normalised with anti-TcHSP70 antibody. The immunoreactive proteins on the membranes were revealed using chemiluminescence. (B-C) Susceptibility to benznidazole (BZ) in BZS (B) and 17WTS (C) *Trypanosoma cruzi* parasites overexpressing or not overexpressing the TceIF5A or eIF5A-S2A mutant protein. Parasites were cultured in the absence or presence of increasing BZ concentrations (2.5 to 20 µM) for 96 h and the percentage of relative growth was determined using a Z1 Coulter Counter. Mean values ± standard deviations from three independent experiments in triplicate are shown. Statistical analysis was carried out using two way analysis of variance (ANOVA) followed by a Bonferroni *post hoc* test. Statistically different values are highlighted as follows: *p < 0.05; **p < 0.01.

The expression of proteins involved in drug resistance or susceptibility could also be affected by eIF5A. In fact, Bao et al.^25^ revealed that overexpression of eIF5A2 reduced doxorubicin sensitivity in colon cancer cells, suggesting that inhibition of eIF5A2 expression may be a potential target to revert resistance in colorectal cancer therapy. Decreased expression of eIF5A was detected in adriamycin-resistant variants of DLKP, a squamous lung cancer cell line.^26^

Our results showed that the protein levels of eIF5A were 2-fold lower in benznidazole-resistant *T. cruzi* populations (BZR and 17LER) than in their respective susceptible counterparts BZS and 17WTS. In addition, the overexpression of eIF5A made these *T. cruzi* populations more susceptible to BZ. Importantly, overexpression of the eIF5A-S2A mutant did not alter the susceptibility these parasites to BZ, indicating that the phosphorylation of the serine residue modulates eIF5A activity, favouring the BZ-susceptibility phenotype. Chung et al.^14^ showed that this serine phosphorylation is important to prevent parasite cell death in the stationary phase, a condition in which nutrients are limiting and parasites might adapt to a new metabolic situation. The eIF5A phosphorylation seems to affect recycling of the protein from elongating polysomes, which could promote a more efficient translation, perhaps of a set of genes. Extensive dephosphorylation occurs in cells in stationary phase, allowing the translation of specific messages that are possibly involved in the stationary stress response. Therefore, we hypothesized that the action of eIF5A in growing parasites might signal a normal situation, which will allow higher susceptibility to BZ damage.

Our previous phosphoproteomic study in *Leishmania braziliensis* demonstrated that the elongation factor 2 (EF2) presented lower abundance in antimony-resistant *L. braziliensis* samples than in the susceptible samples,^27^ suggesting that this protein was dephosphorylated (active state) to regulate the elongation of essential proteins that are crucial to maintain the antimony resistance phenotype.

Alternatively, it is important to consider that the amount of hypusinated eIF5A protein could be directly associated with the levels of polyamines and consequently of trypanothione, a key component in the detoxification BZ.^28^ In addition, Byers et al.^29^ showed that the cytostasis observed in spermidine-deprived cells after treatment with an inhibitor of S-adenosylmethionine decarboxylase was attributed to depletion of hypusine containing-eIF5A. These data revealed that hypusination precursors could affect eIF5A levels directly, suggesting that the decrease of this protein in our BZ-resistant parasites might be a consequence of reduction in spermidine and/or trypanothione levels. Thus, the trypanothione levels in BZ-resistant parasites should be investigated further.

Importantly, several genes are involved in and are differentially expressed during BZ-selected and BZ-natural resistance in *T. cruzi* populations, indicating the multiplicity of mechanisms that contribute to the drug resistance phenotype.^4^ ^,^ ^5^ ^,^ ^30^ In summary, this study revealed a possible link between protein translation and the oxidative metabolism in *T. cruzi* through the participation of eIF5A in the benznidazole-susceptibility mechanism.
